# Systematic Review of Surveys Used to Evaluate Patient and Provider Perspectives on Teleophthalmology

**DOI:** 10.1177/26924366251365510

**Published:** 2025-08-11

**Authors:** Teresa E. Fowler, Gianluca De Leo

**Affiliations:** ^1^ Department of Ophthalmology, Medical College of Georgia at Augusta University, Augusta, Georgia, USA.; ^2^ School of Public Health, Augusta University, Augusta, Georgia, USA.

**Keywords:** teleophthalmology, telemedicine, survey, validation, ophthalmology

## Abstract

**Background:**

Telemedicine has become an important mechanism for delivering health care in the wake of the COVID-19 pandemic. While some medical subspecialties were able to rapidly integrate telecare into their workflow, ophthalmology is one field in which telemedicine has not been as widely adopted. In this systematic review, we analyze published studies assessing patient and provider viewpoints on telemedicine for eye care. Our aim is to understand how adoption of teleophthalmology is being studied and whether definitive conclusions regarding patient and provider perspectives on telecare can be drawn from published literature.

**Methods:**

We performed a systematic PubMed search for studies utilizing surveys to assess patient and provider perspectives regarding remote eye care. Articles were excluded if they were irrelevant to teleophthalmology, did not assess patient or eye care provider perspectives regarding teleophthalmology, assessed specific platforms or aspects of eye care, were reviews without primary data, or if the survey used was not available. The questionnaires from the included articles were analyzed for validation status, subspecialty, question wording, and response format.

**Results:**

The PubMed search returned 92 articles, 22 of which were included in the final dataset after exclusions. Only four studies utilized externally validated questionnaires, although several additional studies were based on validated items. Survey length, wording, and response formatting varied across the studies. These 22 studies contained responses from 3,796 patients and 2,388 eye care professionals, but the lack of standardization between the surveys makes high power conclusions impossible.

**Discussion:**

The results of this review demonstrate a need to develop a standardized and validated survey instrument specifically for assessing teleophthalmology to identify barriers to widespread implementation.

## Introduction

Telemedicine has become an increasingly prevalent means of health care delivery, the adoption and utilization of which has been accelerated by the COVID-19 pandemic. Medicare and Medicaid data show that weekly telehealth visits increased from approximately 13,000 before the COVID-19 pandemic to more than 1.7 million in April 2020,^1^ and in 2020, telemedicine constituted approximately $55.9 billion on the global market.^2^ Telehealth services may be synchronously provided via phone or computer, with the patient and provider linked in real time, or asynchronously with data sent to a remote provider who interprets and completes the visit at a later time. Telemedicine can benefit those with transportation difficulties, chronic illnesses, vision impairment, and other barriers to attending in-person appointments and can expand provider reach so that those in underserved areas can see specialized physicians on time. From the provider’s perspective, telehealth benefits include increased schedule flexibility, patient compliance, increased independence, and reduced expenditure on clinic supplies. Overall, studies report that patient^3–6^ and provider^7,8^ satisfaction are reported to be generally high with telemedicine.

Despite the recent boom in telehealth services and potential benefits, ophthalmology is likely one of the fields utilizing telecare the least.^9^ A thorough ophthalmical examination generally requires some combination of testing visual acuity and intraocular pressures, as well as a physical eye examination using a slit lamp microscope and indirect ophthalmoscopy. Ancillary tests, such as visual fields, optical coherence tomography, gonioscopy, fundus photography, and optical coherence tomography, are common adjuncts. Techniques for obtaining equivalent information virtually have been attempted with varying success, including smartphone apps for the slit lamp examination and home devices for performing ancillary tests.^10–12^

While many groups have successfully provided tele-eye care using these methods, there does not seem to be an overall consensus on the long-term utility and feasibility of incorporating remote care into ophthalmology practices. In this systematic review, we analyze published literature on telemedicine and eye care, specifically including studies that assess patient and provider opinions on this care delivery method. Our aim is to understand how the adoption of teleophthalmology is being studied and whether definitive conclusions regarding patient and provider perspectives on telecare can be drawn from published literature.

## Materials and Methods

We performed a systematic search in PubMed to identify studies assessing patient and provider perspectives on teleophthalmology. The search phrase began with the medical subject heading (MeSH) “telemedicine” followed by Boolean Operator AND to link to “ophthalmolog.” The use of “telemedicine” as a MeSH term allowed us to retrieve all related articles using variations of this term, including “telehealth,” “eHealth,” “virtual medicine,” and “telecare,” among others. The complete list of entry terms included in the “telemedicine” MeSH search can be found on the National Library of Medicine website.^13^

To specifically target studies analyzing direct provider or patient feedback, we included a search of the title or abstract fields for “questionnaire*,” “poll*,” or “surve*.” The entire search phrase used was as follows: (Telemedicine[MeSH Terms]) AND (ophthalmolog*[Title/Abstract]) AND (surve*[Title/Abstract] OR poll*[Title/Abstract] OR questionnaire*[Title/Abstract]). We included all articles published before June 1, 2023, without additional filters. This initial PubMed search yielded 92 articles.

The abstract and methods sections of each article were screened for relevance by author #1 (T.E.F.). “Teleophthalmology” was interpreted broadly by the authors as including any means of remotely delivering eye care using electronic transfer of medical information, including synchronous and asynchronous approaches. Only articles that directly surveyed patients’ or eye care providers’ viewpoints on teleophthalmology were included. The most common reason for exclusion was that the article did not directly assess patient or provider experiences with teleophthalmology (28 articles). The second most common reason for exclusion was that the article assessed a specific application or internet platform rather than teleophthalmology as a general method for care (12 articles). Seven publications were excluded because they surveyed providers from specialties other than ophthalmology, such as emergency providers or general practitioners. We did encounter studies that surveyed eye care professionals other than ophthalmologists, including optometrists and ophthalmical assistants, and decided to include them due to relevance. Six studies were reviews without primary data, three specifically assessed the delivery of newborn eye screens, three were not available in English, two were not relevant to teleophthalmology, and one was a duplicate of an included article. Some articles met multiple of these criteria for exclusion. At the completion of the abstract/methods screen, 62 articles were excluded, with 30 articles remaining in the dataset.

**FIG. 1.**
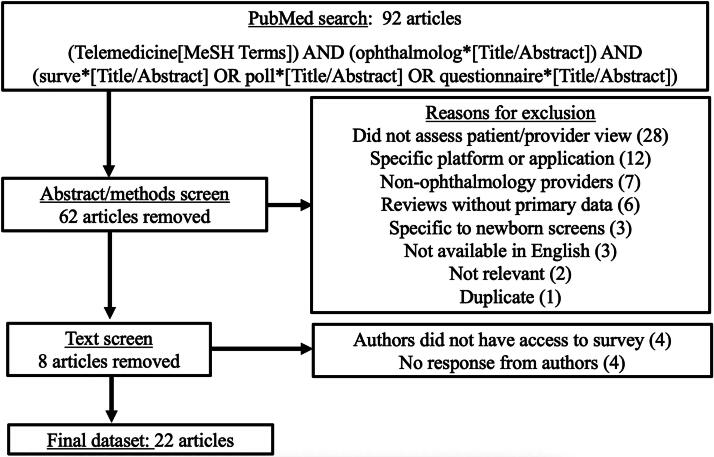
Systematic review article exclusion and inclusion process.

The full text of each selected article was downloaded, and the complete questionnaire was extracted when available. Questionnaires not available in the article’s main body or supplemental materials were requested directly from the authors. Four requests could not be fulfilled as the articles were published several years prior and the authors no longer had access to the original surveys; these articles were excluded as we could not access the specific questions. Four authors did not reply to requests, and thus, these articles were also excluded. After these exclusions, the final dataset included 22 articles. A flow chart demonstrating the article selection process is included in Figure 1. The second author (G.D.L.) independently reviewed the final list of included articles and did not remove or add any additional articles.

The questions used in the original articles were extracted. It was noted whether the questionnaires were validated or nonvalidated, the subject of each question (patient or provider), and the style of the response options (Likert, yes/no, rating scale, multiple choice, open ended). Questions about the demographic characteristics of the respondents were removed such as, “what is your gender?” or “what is your age?”, as these were not relevant to the study objectives. Provider questions asking specific features of their practice which could not be generalized and were not the focus of this study, such as how many years the provider has practiced or the setting of their clinic, were also removed.

Table 1 demonstrates patient-reported questions related to teleophthalmology from each included publication, whereas Table 2 provides corresponding information from the provider’s perspective (Tables 1 and 2). Questions consisting of a single prompt with the ability to mark multiple responses, such as “Which platform do you use? Select all that apply,” were counted as one single question. Questions with a single prompt heading but multiple subquestions with their own answer choices were recorded as separate questions. As an example, Rhodes et al. contained a question reading “Did you know that it is possible to…” with 4 subquestions reading: “(a) obtain a remote medical opinion or diagnosis from a physician via telemedicine technology?”, “(b) receive a medical intervention or treatment supervised remotely by a specialist via telemedicine technology?”, “(c) obtain telephone advice from a health care professional on the appropriate type of service to use?”, and “(d) remotely monitor individual health status via telemedicine technology?” with yes/no answers available for each.^19^ This was counted as 4 separate questions, since each one addressed a unique idea and required a distinct answer. All articles and included questions were cross-checked for accuracy.

**Table 1.** Studies Assessing Patient Perspectives on Teleophthalmology, Including the Study First Author, Subspeciality Where Applicable, Year, Validation Status, Sample Size, and Journal

Study/subspecialty	Use of validated surveys	Sample size	Patient questions	Answer choices
Chen (2022)^14^	Yes: “E-mails containing a validated, 11-item, telehealth satisfaction scale were sent to patients who had at least one video visit within the study period.”	252	How satisfied were you with your overall treatment experience at using telehealth?	(1) poor; (2) fair; (3) good; (4) excellent
			How satisfied were you with the length of time it took to get onto the telehealth platform to see your provider?	(1) poor; (2) fair; (3) good; (4) excellent
			How satisfied were you with the voice quality of the equipment?	(1) poor; (2) fair; (3) good; (4) excellent
			How satisfied were you with the visual quality of the equipment?	(1) poor; (2) fair; (3) good; (4) excellent
			How satisfied were you with your personal comfort in using the telehealth system?	(1) poor; (2) fair; (3) good; (4) excellent
			How satisfied were you with the length of time with the ophthalmologist?	(1) poor; (2) fair; (3) good; (4) excellent
			How satisfied were you with the explanation of your treatment by the ophthalmologist?	(1) poor; (2) fair; (3) good; (4) excellent
			How satisfied were you with the thoroughness, carefulness, and skillfulness of the ophthalmologist?	(1) poor; (2) fair; (3) good; (4) excellent
			How satisfied were you with the courtesy, respect, sensitivity, and friendliness of the ophthalmologist?	(1) poor; (2) fair; (3) good; (4) excellent
			How well was your privacy respected?	(1) poor; (2) fair; (3) good; (4) excellent
			How well did the staff answer your questions about the equipment?	(1) poor; (2) fair; (3) good; (4) excellent
Conway (2021)^15^ Neuro-ophthalmology	No	159	How easy was it to understand the instructions to prepare for your virtual health visit?	(a) Very easy; (b) Somewhat easy; (c) Somewhat difficult; (d) Very difficult
			Do you feel that the virtual health visit satisfied your needs, or did it feel like you still needed to be seen in-person?	(a) Virtual health visit satisfied my needs; (b) Felt like I still needed to be seen in-person
			Is there anything that would have helped you to better prepare for your virtual health visit?	(a) No; (b) Yes; if yes, what would have helped?
			Were there any particular aspects of the virtual health visit that you found challenging?	(a) No; (b) Yes; if yes, what aspects did you find challenging?
			How comfortable were you with the virtual health visit compared with an in-person visit regarding asking questions about your health?	(a) Very comfortable; (b) Somewhat comfortable; (c) Somewhat uncomfortable; (d) Very uncomfortable
Gerbutavicius (2021)	No	29	How would you rate the allocation of your video consultation appointment?	1–6 scale
			Did your video consultation take place?	(a) Yes; (b) No
			How easy did you find it to access the video consultation?	1–6 scale
			How stable was the connection in terms of sound and image quality?	1–6 scale
			How would you rate your overall experience with video-telephony?	1–6 scale
			Would you recommend a video consultation to someone else?	(a) Yes; (b) No
			Did you hold the video consultation via your cell phone, tablet, laptop, or PC?	Open ended
			If you used your cell phone or tablet for the video consultation, what operating system does your device use? (iOS/Android)	Open ended
Kalra (2021)	No	56	On a scale of 1–5, 1 being worse and 5 being resolving/resolved, how are your symptoms since the encounter?	(1) Worse–(5) Resolved
			On a scale of 1–5, 1 being not at all helpful and 5 being very helpful, how helpful was the encounter in meeting your needs?	(1) Not helpful at all–(5) Very helpful
			On a scale of 1–5, 1 being not at all convenient and 5 being very convenient, how convenient was the encounter?	(1) Not at all convenient–(5) Very convenient
			On a scale of 1–5, 1 being much less than in-person and 5 being much more than in-person, how would you rate the safety of a virtual encounter to coming to the hospital in-person given the COVID crisis?	(1) Much less than in-person–(5) Much more than in-person
Kurji (2013)^16^ Diabetic patients	No	57	The nurse was knowledgeable and explained clearly to me the reasons why teleophthalmology was being used for my diabetic screening.	(1) Strongly disagree; (2) Disagree; (3) Neither agree nor disagree; (4) Agree; (5) Strongly agree
			I spent less time waiting at my teleophthalmology diabetic screening than when I had to see the eye doctor.	(1) Strongly disagree; (2) Disagree; (3) Neither agree nor disagree; (4) Agree; (5) Strongly agree
			Compared to seeing the eye doctor, the use of teleophthalmology was much more convenient for me	(1) Strongly disagree; (2) Disagree; (3) Neither agree nor disagree; (4) Agree; (5) Strongly agree
			The information I received at my teleophthalmology screening was the same as the information I got when I saw the eye doctor	(1) Strongly disagree; (2) Disagree; (3) Neither agree nor disagree; (4) Agree; (5) Strongly agree
			I like the idea of only seeing the eye doctor when it is necessary (i.e., if therapy or new medicine is needed).	(1) Strongly disagree; (2) Disagree; (3) Neither agree nor disagree; (4) Agree; (5) Strongly agree
			I like the idea of being able to see the inside of my eye (retina) as the eye doctor sees it	(1) Strongly disagree; (2) Disagree; (3) Neither agree nor disagree; (4) Agree; (5) Strongly agree
			Did you pick up your report from the eye clinic? If you did not pick up your picture, why not?	(a) Yes; (b) No
			When I came to pick up the report, I understood the contents clearly as explained by the nurse	(1) Strongly disagree; (2) Disagree; (3) Neither agree nor disagree; (4) Agree; (5) Strongly agree
			I was totally happy with the use of teleophthalmology for my diabetic screening.	(1) Strongly disagree; (2) Disagree; (3) Neither agree nor disagree; (4) Agree; (5) Strongly agree
			For my next diabetic screening visit, I would prefer to use teleophthalmology rather than having an appointment to see the eye doctor	(1) Strongly disagree; (2) Disagree; (3) Neither agree nor disagree; (4) Agree; (5) Strongly agree
Martin (2020)^17^ Diabetic patients	No	114	Facility to make an appointment at the health care center to conduct the test.	(0) Bad; (1) Average; (2) Good; (3) Very good
			Time devoted to conduct the test.	(0) Bad; (1) Average; (2) Good; (3) Very good
			Explanations received before conducting the test.	(0) Bad; (1) Average; (2) Good; (3) Very good
			Explanations received during the test.	(0) Bad; (1) Average; (2) Good; (3) Very good
			Explanations received after conducting the test.	(0) Bad; (1) Average; (2) Good; (3) Very good
			The feeling of being in good hands during the test.	(0) Bad; (1) Average; (2) Good; (3) Very good
			Treatment received during the test.	(0) Bad; (1) Average; (2) Good; (3) Very good
			Waiting time to receive the results.	(0) Bad; (1) Average; (2) Good; (3) Very good
			Tolerance to pupil dilation.	(0) Bad; (1) Average; (2) Good; (3) Very good
			Score, on a scale from 0 to 10, for degree of general satisfaction with the activity.	0–10 scale
			Willingness to continue having the check-up at the health care center.	(a) Yes; (b) No
Newman-Casey (2021)^18^	No	1720	On a scale from 1–10 where 1 is completely dissatisfied and 10 is completely satisfied, how would you rate your satisfaction with your eye care from Kellogg during the coronavirus epidemic, whether your care has been deferred, you were seen in-person, or whether you were seen though a video or phone visit?	1–10 scale
			How did you feel about having your visits be virtual (over the phone or internet) during the coronavirus pandemic?	
Rhodes (2019)^19^ Glaucoma	Yes: “Enrolled patients were administered a Life Space questionnaire (LSQ), scored 0–9, and Preferences for Telemedicine questionnaire (PTQ), a Likert scale validated tool.”	110	Have you ever heard about telemedicine?	(a) Yes; (b) No
			Did you know that it is possible to obtain a remote medical opinion or diagnosis from a physician via telemedicine technology?	(a) Yes; (b) No
			Did you know that it is possible to receive a medical intervention or treatment supervised remotely by a specialist via telemedicine technology?	(a)Yes; (b) No
			Did you know that it is possible to obtain telephone advice from a health care professional on the appropriate type of service to use?	(a) Yes; (b) No
			Did you know that it is possible to remotely monitor individual health status via telemedicine technology?	(a) Yes; (b) No
			To what extent do you agree with these statements: Telemedicine could provide continuing education to health care professionals	1. Strongly agree; 2. Somewhat agree; 3. Neutral; 4. Somewhat disagree; 5. Strongly disagree
			To what extent do you agree with these statements: Telemedicine could contribute to reducing health care system costs	1. Strongly agree; 2. Somewhat agree; 3. Neutral; 4. Somewhat disagree; 5. Strongly disagree
			To what extent do you agree with these statements: Telemedicine could improve access to health care services for people in remote regions	1. Strongly agree; 2. Somewhat agree; 3. Neutral; 4. Somewhat disagree; 5. Strongly disagree
			To what extent do you agree with these statements: Telemedicine could provide better quality health care services for the population as a whole	1. Strongly agree; 2. Somewhat agree; 3. Neutral; 4. Somewhat disagree; 5. Strongly disagree
			To what extent are you concerned about Confidentiality of personal information in telemedicine transmissions	1. Not concerned at all; 2. Slightly concerned; 3. Neutral; 4. Very concerned; 5. Extremely concerned
			To what extent are you concerned about absence of direct contact with health care providers during a telemedicine consultation	1. Not concerned at all; 2. Slightly concerned; 3. Neutral; 4. Very concerned; 5. Extremely concerned
			To what extent are you concerned about Legal responsibility in the case of medical errors occurring during telemedicine interventions	1. Not concerned at all; 2. Slightly concerned; 3. Neutral; 4. Very concerned; 5. Extremely concerned
			To what extent do you agree with these statements: You would be willing to receive a remote medical diagnosis from a physician via telemedicine technology.	1. Strongly agree; 2. Somewhat agree; 3. Neutral; 4. Somewhat disagree; 5. Strongly disagree
			To what extent do you agree with these statements: You would be willing to receive a remote medical intervention or treatment from a physician via telemedicine technology.	1. Strongly agree; 2. Somewhat agree; 3. Neutral; 4. Somewhat disagree; 5. Strongly disagree
Sieberer (2022)^20^	No	104	Did you feel you had sufficient explanation and instruction on the approach of the consultation?	(1) Very poor/strongly disagree; (2) Poor/disagree; (3) Good/agree; (4) Very good/strongly agree
			Would you have wished for a more detailed instruction on the line of action?	(1) Very poor/strongly disagree; (2) Poor/disagree; (3) Good/agree; (4) Very good/strongly agree
			Did you feel you were able to give your history adequately?	(1) Very poor/strongly disagree; (2) Poor/disagree; (3) Good/agree; (4) Very good/strongly agree
			Did you feel safe throughout the consultation and examination?	(1) Very poor/strongly disagree; (2) Poor/disagree; (3) Good/agree; (4) Very good/strongly agree
			Did you feel that your examination has been adequate?	(1) Very poor/strongly disagree; (2) Poor/disagree; (3) Good/agree; (4) Very good/strongly agree
			Did you feel you had enough room to express your concerns and discuss your diagnosis and treatment options?	(1) Very poor/strongly disagree; (2) Poor/disagree; (3) Good/agree; (4) Very good/strongly agree
			Were you surprised with how the consultation took place today?	(1) Very poor/strongly disagree; (2) Poor/disagree; (3) Good/agree; (4) Very good/strongly agree
			How was your overall experience of your visit to the Eye clinic?	(1) Very poor/strongly disagree; (2) Poor/disagree; (3) Good/agree; (4) Very good/strongly agree
			I liked…/Positive comments	Open ended
			I did not like.../Areas of improvement	Open ended
Staffieri (2021)^21^ Pediatric	No	89	Thinking about your recent telehealth consultation, how would you rate your overall satisfaction?	(1) Very unsatisfied; (2) Unsatisfied; (3) Neutral; (4) Satisfied; (5) Very satisfied
			Thinking about your recent experience, would you consider further telehealth consultations in the future?	(a) Yes; (b) Maybe; (c) No
			We would like to adapt this service to be of maximum benefit. Do you have any suggestions for us to improve this service?	Open ended
			Do you have any other comments, questions, or concerns?	Open ended
Summers (2022)^22^	No	817	How likely would you be to recommend this facility to your family and friends, where a score of 0 being not at all likely and 10 being extremely likely?”	Score 0–10
			How likely would you be to recommend this provider to your family and friends, where a score of 0 being not at all likely and 10 being extremely likely?	Score 0–10
			For virtual/phone visits only: On a scale of 1 to 10, how would you rate how easy it was to connect to your phone or virtual visit?	Score 1–10
			For virtual/phone visits only: On a scale of 1 to 10, how would you rate the quality of the technology used for your phone or virtual visit?	Score 1–10
Sung (2023)^23^	No	98	How easy to communicate with the ophthalmologist via teleophthalmology system?	(1) Very poor; (2) Poor; (3) Fair; (4) Good; (5) Very Good
			Did teleophthalmology reduce the travel time to a hospital or ophthalmical clinic?	(1) Very poor; (2) Poor; (3) Fair; (4) Good; (5) Very Good
			Did teleophthalmology save the cost of medical expenses?	(1) Very poor; (2) Poor; (3) Fair; (4) Good; (5) Very Good
			Did teleophthalmology provide the deserved medical care?	(1) Very poor; (2) Poor; (3) Fair; (4) Good; (5) Very Good
			Would you utilize teleophthalmology system in the future again?	(1) Very poor; (2) Poor; (3) Fair; (4) Good; (5) Very Good
			How satisfied with real-time doctor consultation are you?	(1) Very poor; (2) Poor; (3) Fair; (4) Good; (5) Very Good
			How useful was the suggestion from the ophthalmologist via teleophthalmology?	(1) Very poor; (2) Poor; (3) Fair; (4) Good; (5) Very Good
			The overall satisfaction of teleophthalmology	(1) Very poor; (2) Poor; (3) Fair; (4) Good; (5) Very Good
Tan (2013)^24^	No	30	Similar to face-to-face consultation	(a) Yes; (b) No
			Achieved purpose of consultation	(a) Yes; (b) No
			Acceptable waiting time	(a) Yes; (b) No
			Preferred choice for future ophthalmical consultations	(a) Yes; (b) No
			Would recommend teleophthalmology consultation to friends and relatives	(a) Yes; (b) No
			Satisfaction with teleophthalmology workflow and consultation	(a) Very satisfied; (b) Satisfied; (c) Neutral; (d) Dissatisfied; (e) Very dissatisfied
Valikodath (2017)^25^ Diabetic patients	Yes: “The RAND tele-ophthalmology questionnaire was used to assess information that was specific to people with DM, including duration of diabetes.33 The survey utilized validated instruments to assess access to medical care.”	97	Have you ever heard of telemedicine or teleophthalmology before? Yes/No	(1) Yes; (2) No
			What have you heard about telemedicine or teleophthalmology?	Open ended
			I would be willing to receive my eye examinations this way.	(a) Strongly agree; (b) Agree; (c) Uncertain; (d) Disagree; (e) Strongly disagree
			I worry about whether my information would be kept private if sent by computer from the primary doctor to the eye specialist.	(a) Strongly agree; (b) Agree; (c) Uncertain; (d) Disagree; (e) Strongly disagree
			I believe this method of eye examination would be more convenient than going to a separate eye appointment.	(a) Strongly agree; (b) Agree; (c) Uncertain; (d) Disagree; (e) Strongly disagree
			I am concerned about whether the images would be read correctly over the computer.	(a) Strongly agree; (b) Agree; (c) Uncertain; (d) Disagree; (e) Strongly disagree
			I would like to get the results of the eye examination immediately.	(a) Strongly agree; (b) Agree; (c) Uncertain; (d) Disagree; (e) Strongly disagree
			I would like this method more if I could communicate with the eye specialist at the time the pictures were being taken (such as over the phone or through a video image with sound).	(a) Strongly agree; (b) Agree; (c) Uncertain; (d) Disagree; (e) Strongly disagree
			I would not be willing to receive my eye examinations in this way.	(a) Strongly agree; (b) Agree; (c) Uncertain; (d) Disagree; (e) Strongly disagree
			I would miss interacting with my eye doctor in person.	(a) Strongly agree; (b) Agree; (c) Uncertain; (d) Disagree; (e) Strongly disagree

Questions relevant to teleophthalmology were extracted from each study, along with the answer choices provided.

DM, diabetes mellitus; RAND, RAND corporation.

**Table 2.** Studies Assessing Provider Perspectives on Teleophthalmology, with the Publication Information, Sample Size, and Relevant Questions with Answer Choices Extracted

Study/subspecialty	Use of validated surveys	Sample size	Provider questions	Answer choices
Agarwal (2021)^26^	Yes: “The face validity was ascertained by four independent senior ophthalmologists from tertiary eye care hospitals. Pilot testing of the questionnaire was done among 20 registered ophthalmologists.”	1026	What is your experience regarding utility of teleophthalmology in your practice during lockdown period and after? (Multiple options can be marked)	(a) No experience with teleophthalmology; (b) It did no match my expectation and not many of my patients were benefitted; (c) It benefitted my patients, and I am satisfied; (d) It was billed similar to my OPD face-to-face consultation charges; (e) It was billed lower than my usual consultation charges; (f) It was not billed
Assayag (2021)^27^ Oculoplastic	No	70	In your opinion, is telemedicine an effective tool for oculoplastic consultations?	(a) Yes; (b) No
			Were you utilizing telemedicine in your practice prior to the COVID-19 outbreak?	(a) Yes; (b) No
			Given the current status of COVID-19 in your country and setting, do you feel sufficiently protected in terms of personal protective equipment and implementation of COVID-19 guidelines?	(a) Yes; (b) No
			Since the COVID-19 outbreak, was your outpatient clinic activity reduced or limited to urgent cases only?	(a) Yes; (b) No
			Since the COVID-19 outbreak, have you incorporated telemedicine into your clinical practice?	(a) Yes; (b) No
			Do you expect telemedicine to be in greater use in your practice after the COVID-19 pandemic subsides?	(a) Yes; (b) No
Cohen (2022)^28^ Retina	No	361	Did you perform any telemedicine visits (via phone or video) prior to March 1 of 2020?	(a) Yes; (b) No
			(If yes) how many weekly visits were performed over telemedicine?	(a) 0–5; (b) 5–10; (c) 10–15; (d) >15
			Since March 1, 2020, have you performed any telemedicine visits (via phone or video)?	(a) Yes; (b) No
			(If yes) how many weekly visits were performed over telemedicine?	(a) 0–5; (b) 5–10; (c) 10–15; (d) >15
			What modality do you use for telemedicine?	(a) Phone only; (b) Video only; (c) Phone and video
			Which platforms have you used for telemedicine?	(a) Facetime; (b) EMR based; (c) Zoom; (d) Google; (e) Other (free text)
			Do your patients have access to home-based or remote-based fundus imaging (either color photography or OCT) that are available during your telemedicine consultation?	(a) Yes; (b) No
			What resources have you utilized most to implement your telemedicine guidelines/protocols? (Please select all that apply)	(a) AAO; (b) ASRS; (c) AMA; (d) Local institutional policies; (e) We have no telemedicine guidelines/protocol; (f) Other (free text)
			In its current form, I find telemedicine visits to be an acceptable form of evaluation for retina patients in the right clinical scenario.	(a) Strongly agree; (b) Somewhat agree; (c) Neither agree nor disagree; (d) Somewhat disagree; (e) Strongly disagree
			If remote-based fundus imaging was available, telemedicine visits would be an acceptable replacement for in-person visits in the right clinical scenario.	(a) Strongly agree; (b) Somewhat agree; (c) Neither agree nor disagree; (d) Somewhat disagree; (e) Strongly disagree
			Which of the following do you think are the barriers to successful implementation of telemedicine in the field of retina? Concern that pathology or diagnosis will be missed without in-person examination	(a) Strongly agree; (b) Somewhat agree; (c) Neither agree nor disagree; (d) Somewhat disagree; (e) Strongly disagree
			Inability to obtain remote or home-based fundus imaging	(a) Strongly agree; (b) Somewhat agree; (c) Neither agree nor disagree; (d) Somewhat disagree; (e) Strongly disagree
			Inability to obtain remote or home-based OCT imaging	(a) Strongly agree; (b) Somewhat agree; (c) Neither agree nor disagree; (d) Somewhat disagree; (e) Strongly disagree
			Concern about legal liability	(a) Strongly agree; (b) Somewhat agree; (c) Neither agree nor disagree; (d) Somewhat disagree; (e) Strongly disagree
			Technological requirements (both access to and comfort level with)	(a) Strongly agree; (b) Somewhat agree; (c) Neither agree nor disagree; (d) Somewhat disagree; (e) Strongly disagree
			Low level of reimbursement for overall time spent	(a) Strongly agree; (b) Somewhat agree; (c) Neither agree nor disagree; (d) Somewhat disagree; (e) Strongly disagree
			Concerns regarding data security and HIPAA violations	(a) Strongly agree; (b) Somewhat agree; (c) Neither agree nor disagree; (d) Somewhat disagree; (e) Strongly disagree
			If you currently use telemedicine, which of the following best agrees with your planned use of it in the future?	(a) I will continue during and after COVID-19; (b) I will continue as long as in-person visits are made more difficult/dangerous due to COVID-19, then I will stop; (c) I do not perform telemedicine visits; (d) Other (free text)
Conway (2021)^15^ Neuro-ophthalmology	No	157	Were you able to complete an examination as part of the virtual health visit that provided enough information for medical decision-making?	(a) Yes; (b) No; if no, what aspects were inadequate and/or needed to be determined or performed in-person?
			What parts of the examination did you find surprisingly easy to gather useful information from? Please circle all that apply.	(a) Visual acuity; (b) Amsler grids; (c) Color plates (Ishihara); (d) Red desaturation; (e) Pupils; (f) Visual fields; (g) Range of eye movements; (h) Ocular alignment; (i) Saccade speed and accuracy; (j) Smooth pursuit; (k) VOR; (l) VOR suppression; (m) Convergence; (n) OKN; (o) Ocular oscillations (nystagmus and saccadic intrusions)
			Are there any aspects that could help enhance the quality of the information obtained virtually? (positioning of the phone cameras or lighting, for example)	(a) No; (b) Yes; if yes, what could have helped?
			Approximately how many virtual health visits have you performed to date using the current platforms?	Open ended
De Lott (2021)	No	88	Before the coronavirus epidemic, did you provide any of the following telemedicine services? (check all that apply)	(a) Phone visits; (b) Video visits; (c) Interprofessional e-consultations; (d) none
			Since the coronavirus epidemic began, how many times have you conducted consults with other health care providers that included photographs or videos provided in person, through e-mail, or online?	(a) Never; (b) 1–2 times; (c) 3–10 times; (d) ≥11 times
			Since the coronavirus epidemic began, how many times have you received photographs from patients through e-mail or online?	(a) Never; (b) 1–2 times; (c) 3–10 times; (d) ≥11 times
			Since the coronavirus epidemic began, how many times have you conducted video visits with patients?	(a) Never; (b) 1–2 times; (c) 3–10 times; (d) ≥11 times
			Since the coronavirus epidemic began, how many times have you conducted phone visits with patients?	(a) Never; (b) 1–2 times; (c) 3–10 times; (d) ≥11 times
			Based on your experience with telemedicine since the coronavirus epidemic began, how would you describe your confidence in using remote screening for eye care?	(a) Not confident at all; (b) Somewhat confident; (c) Confident; (d) Extremely confident; (e) N/A
			Since the coronavirus epidemic began, how do you feel about telemedicine utilization in ophthalmology?	(a) Highly underutilized; (b) Somewhat underutilized; (c) Utilized appropriately; (d) Somewhat overutilized; (e) Highly overutilized
			How likely are you to continue to provide eye telemedicine services (video visits, phone visits, e-consultations) for the next 1 year?	(a) Unlikely; (b) Somewhat unlikely; (c) Unsure; (d) Somewhat likely; (e) Likely
Faes (2020) Retina	No	214	Did your institution provide virtual or remote assessments for the triage of patients (i.e., telephone-triage to decide who needs to be seen by an ophthalmologist BEFORE THE PANDEMIC?	(a) Yes; (b) No; (c) Don’t know; (d) Other
			Were there any approaches to “smart history-taking” in your clinic? (i.e., technicians supported by a decision-tree, smartphone apps, etc.) BEFORE the pandemic?	(a) Yes; (b) No; (c) Don’t know; (d) Other
			Did your institution provide virtual or remote consultations (i.e., via telephone, video, remote assessment of multimodal imaging, movie-based patient education, virtual second-opinion platforms) BEFORE THE PANDEMIC?	(a) Yes; (b) No; (c) Don’t know; (d) Other
			Did your institution have established virtual clinics or teleophthalmology services BEFORE THE PANDEMIC?	(a) Yes; (b) No; (c) Don’t know; (d) Other
			Has an electronic referral platform been used in your institution (i.e., doctors from primary or secondary care referring to tertiary care) BEFORE THE PANDEMIC?	(a) Yes; (b) No; (c) Don’t know; (d) Other
			Has your institution regularly used remote assessment of multimodal imaging to substitute slit-lamp investigation BEFORE THE PANDEMIC?	(a) Yes; (b) No; (c) Don’t know; (d) Other
			Did your institution use automated imaging analyses- or decision-making tools and/or tools for automated triage of patients BEFORE THE PANDEMIC?	(a) Yes; (b) No; (c) Don’t know; (d) Other
			How often was teleophthalmology used in your institution BEFORE THE PANDEMIC?	6 point scale from “Never” to “Frequently”
			BEFORE THE PANDEMIC: Did you think teleophthalmology was useful to deliver eye care to your patients?	5 point scale from “Not useful at all” to “Very useful”
			Is your institution CURRENTLY doing teleophthalmology (virtual triage/ consultations/ referrals)?	(a) Yes; (b) No; (c) Don’t know; (d) Other
			Is your institution CURRENTLY using teleophthalmology to reduce contact time with patients (i.e., remote history taking via video call from a separate room before a face-to-face assessment via slit-lamp)?	(a) Yes; (b) No; (c) Don’t know; (d) Other
			Is your institution CURRENTLY using “imaging only” approaches to substitute the slit-lamp investigation in your institution?	(a) Yes; (b) No; (c) Don’t know; (d) Other
			“If teleophthalmology was used DURING THE PANDEMIC in your institution, what would you use it for?”	(a) Counseling; (b) Triage; (c) Therapy instructions; (d) Follow-up
			Do you think the experience you are making with teleophthalmology during the pandemic will sustainably change the way you practice ophthalmology?	(a) Yes; (b) No; (c) Don’t know; (d) Other
			What sort of anatomical home monitoring did your institution use in patients with retinal disease BEFORE THE PANDEMIC? Please select all that apply:	(a) Paper-based (i.e., Amsler Grid); (b) Smartphone-based (i.e., Alleye); (c) Hardware-based (i.e., Preferential hyperacuity perimetry); (d) None; (e) Don’t know; (f) Other
			Which visual functions did your institution home monitor BEFORE THE PANDEMIC? Please select all that apply:	(a) Visual acuity; (b) Hyperacuity/metamorphopsia/scotoma; (c) Contrast sensitivity; (d) None; (e) Don’t know; (f) Other
			For which retinal diseases did your institution home monitor visual function BEFORE THE PANDEMIC? Please select all that apply:	(a) Age-related macular degeneration; (b) Diabetic macular edema; (c) Cystoid macular edema (i.e., due to vein occlusion or Irvine–Gass Syndrome); (d) None; (e) Don’t know; (f) Other
			What sort of anatomical home monitoring did your institution use in patients with retinal disease BEFORE THE PANDEMIC? Please select all that apply:	(a) Home OCT (i.e., Notal Vision); (b) Fundus photography (i.e., smartphone-based: Peek Vision); (c) None; (d) Don’t know; (e) Other
			BEFORE THE PANDEMIC: Did you think home monitoring was useful to deliver retinal care?	(a) Yes; (b) No; (c) Don’t know; (d) Other
			How did the use of home monitoring change in your institution DURING THE PANDEMIC?	(a) Counseling; (b) Triage; (c) Therapy instructions; (d) Follow-up
			Do you think the experience you are making with home monitoring during the pandemic will sustainably change the way you practice ophthalmology?	(a) Yes; (b) No; (c) Don’t know; (d) Other
			What other types of decentralized patient care did your institution provide BEFORE THE PANDEMIC? Please select all that apply:	“(a) Screening for retinal disease in nonmedical settings (i.e., nursing homes, pharmacies, etc.); (b) Intravitreal injections in nonmedical settings (i.e., in patient homes); (c) None; (d) Don’t know; (e) Other”
			How comfortable do you or would you feel to provide decentralized patient care in nonmedical settings?	5-point scale from “Uncomfortable” to “Very comfortable”
			“Do you think the experience you are making with decentralized patient care during the pandemic will sustainably change the way you practice ophthalmology?”	(a) Yes; (b) No; (c) Don’t know; (d) Other
			“What sort of barriers challenged the adoption of teleophthalmology, home monitoring, and decentralized patient care BEFORE THE PANDEMIC in your institution?”	(a) Reimbursement; (b) Patient’s acceptance; (c) Level of infrastructure; (d) Doctor’s acceptance; (e) Don’t know; (f) No barriers; (g) Other
			Are the following services CURRENTLY reimbursed in your institution?	(a) Teleophthalmology; (b) Home monitoring; (c) Decentralized patient care (other); (d) Yes; (e) No; (f) Don’t know
			In your opinion, how could teleophthalmology, home monitoring, and other types of decentralized patient care contribute to tackle the challenges of retinal-care delivery DURING THE PANDEMIC?	Open ended
			Which patients with retinal disease are CURRENTLY seen face-to-face and/or treated in Retina and Uveitis clinics in your institution? Please select all that apply:	(a) Wet AMD; (b) Retinal detachments; (c) Retinal tears; (d) Proliferative diabetic retinopathy; (e) Diabetic macular edema; (f) Uncontrolled uveitis; (g) Retinal vein occlusion; (h) All first-time referrals; (i) Myopic CNV; (j) ROP (screening); (k) F-2-F consultation; (l) Treated
			In your opinion, which patients with retinal/uveitic disease are at risk for irreversible vision loss due to impeded care delivery DURING THE PANDEMIC?	(a) Wet AMD; (b) Retinal detachments; (c) Retinal tears; (d) Proliferative diabetic retinopathy; (e) Diabetic macular edema; (f) Uncontrolled uveitis; (g) Retinal vein occlusion with neovascularizations; (h) First-time referrals; (i) Myopic maculopathy, including choroidal neovascularizations; (j) Retinopathy of prematurity (incl screening); (k) None; (l) Other
			How can future service extensions involving teleophthalmology, home monitoring, and other types of decentralized patient care avoid that patients face irreversible vision loss during ANOTHER PANDEMIC?	Open ended
			Do you think that the experience gained during the pandemic (with teleophthalmology, home monitoring, or other types of decentralized patient care) will be useful for routine care in the future?”	Open ended
			Please state any ongoing projects within teleophthalmology, home monitoring, or other types of decentralized patient care in your country you are aware of that resulted from the challenges posed by the pandemic or write “None.”	Open ended
			We may have missed to ask an important question. Please add any further comments you have below or write ““None”:”	Open ended
Karslioglu (2021)	No	113	Do you think that online video calls/examination applications are beneficial to the health service received by the patient?	(a) Strongly disagree; (b) Disagree; (c) I don’t know; (d) Agree; (e) Strongly agree
			Do you use an online video call/examination application with your patients through your institution?	(a) Yes; (b) No
Martin (2020)^17^ Diabetic patients	No	10	I am aware of the importance that the screening program has for the early diagnosis of DR and the prevention of blindness in diabetic patients.	(1) Totally disagree; (2) Disagree; (3) Indifferent; (4) Agree; (5) Totally agree
			I consider I have received enough theoretical and practical training to perform the activity.	(1) Totally disagree; (2) Disagree; (3) Indifferent; (4) Agree; (5) Totally agree
			I find it easy to handle the retinograph and the computing equipment that we use in this program.	(1) Totally disagree; (2) Disagree; (3) Indifferent; (4) Agree; (5) Totally agree
			I consider that the fundus images I can get have enough quality to be evaluated at the reading center.	(1) Totally disagree; (2) Disagree; (3) Indifferent; (4) Agree; (5) Totally agree
			I consider the patients are well informed about the program when they come to get their tests done	(1) Totally disagree; (2) Disagree; (3) Indifferent; (4) Agree; (5) Totally agree
			I feel confident in front of the patients when performing this activity.	(1) Totally disagree; (2) Disagree; (3) Indifferent; (4) Agree; (5) Totally agree
			I feel I have enough support from the professionals in charge of the implementation of the screening program.	(1) Totally disagree; (2) Disagree; (3) Indifferent; (4) Agree; (5) Totally agree
			I am afraid of the pupil dilation procedure of patients.	(1) Totally disagree; (2) Disagree; (3) Indifferent; (4) Agree; (5) Totally agree
			The activity allows me to improve the doctor–patient relationship with the screening program users	(1) Totally disagree; (2) Disagree; (3) Indifferent; (4) Agree; (5) Totally agree
			I find it easy to combine this activity and my daily care-providing work	(1) Totally disagree; (2) Disagree; (3) Indifferent; (4) Agree; (5) Totally agree
			Score, on a scale from 0 to 10, your degree of general satisfaction with the activity.	0–10 scale
			Would you like to keep performing this activity?	(a) Yes; (b) No
Mercer (2022)^29^	No	73	Based on your experiences during the pandemic, how much of a role do you think there is for teleophthalmology in your regular practice?	(a) No role; (b) Minimal role; (c) Modest role; (d) Large role
			Do you think you will offer teleophthalmology visits once regular face-to-face clinics resume?	(a) Yes, I plan to offer regular teleophthalmology visits; (b) Yes, but only in exceptional situations; (c) No, I do not expect to use teleophthalmology; (d) Don’t know
			Tell me about your experience using teleophthalmology for patient care during the COVID-19 pandemic.	Open ended
			What did you find useful about doing teleophthalmology visits?	Open ended
			What did you find difficult about doing teleophthalmology visits?	Open ended
			Now that regular face-to-face clinics have resumed, how are you using teleophthalmology in your practice?	Open ended
Moss (2020)^30^ Neuro-Ophthalmology	No	208	Prior to the COVID-19 pandemic, were you utilizing live video (synchronous) telemedicine visits in your practice? (For U.S. members, use reference date of before March 1, 2020)	(a) Yes; (b) It was available to me, but I did not use it; (c) No
			Prior to the COVID-19 pandemic, approximately how many video visits did you perform per week? (For U.S. members, use reference date of before March 1, 2020)	(a) None; (b) 1–10 visits; (c) 11–20 visits; (d) 21–30 visits; (e) 31–40 visits; (f) >40
			Presently, do you use live video (synchronous) telemedicine visits in your practice?	(a) Yes. I utilize live video telemedicine visits in my practice; (b) Live video telemedicine is available in my practice, but I do not utilize it; (c) No, but I would like to; (d) No and I am not interested in doing this
			What live video telemedicine platform system do you use? Select as many as apply	(a) Telemedicine platform integrated with my EMR; (b) Zoom; (c) Doxy.me; (d) Webex; (e) Facetime; (g) Skype; (h) Doximity video; (i) Other (fill in)
			After the COVID-19 pandemic, approximately how many video visits do you perform per week? (For U.S. members, use reference date of after March 1, 2020)	(a) None; (b) 1–10 visits; (c) 11–20 visits; (d) 21–30 visits; (e) 31–40 visits; (f) >40
			Assuming reimbursement continues to cover live video (synchronous) telemedicine visits, do you plan to continue this after the COVID-19 public health emergency subsides?	(a) Yes; (b) No; (c) Undecided
			Which of the following have you experienced due to the implementation of live video (synchronous) telemedicine visits in neuro-ophthalmology? (check all that apply)	(a) Increased access to care for patients; (b) Improved interprofessional communications; (c) Continuity of care for patients unable to be seen in the office; (d) Decreased overhead expenses; (e) Increased appointment efficiency for patient (no travel rooming/screening/check in); (f) Increased appointment efficiency for doctor; (g) Increased clinical volume; (h) Improved patient–physician relationship; (i) Other (fill in)
			What do you perceive to be barriers for you in the implementation of live video (synchronous) telemedicine visits in neuro-ophthalmology? (check all that apply)	(a) Difficulty with implementation (e.g., finding appropriate technology services, integration with EHR, learning curve for providers and staff, etc.); (b) Institutional “buy-in”; (c) Reimbursement concerns; (d) Medical liability concerns; (e) Patient privacy concerns; (f) Disruption to personal satisfaction with existing practice model without telemedicine; (g) Limitations in types and quality of data collected (including examination limitations); (h) Other (fill in)
			For each condition, indicate if you perceive a live synchronous video visit would be suitable to evaluate or triage the condition to the appropriate level of care:	(a) Anisocoria; (b) Binocular double vision; (c) Cranial nerve palsy(ies); (d) Eye pain with normal eye examination; (e) Migraine with aura; (f) Nonarteritic anterior ischemic optic neuropathy; (g) Possible arteritic ischemic optic neuropathy; (h) Nystagmus; (i) (Ocular) myasthenia gravis; (j) Optic atrophy; (k) Optic neuritis with appropriate fundus photographs or OCT/visual field and MR imaging available for you to review; (l) Pituitary tumor with visual field and MR sellar imaging available for your interpretation; (m) Positive visual phenomenon (e.g., visual snow) with normal imaging; (n)Pseudotumor cerebri/IIH with available fundus photographs or OCT and visual fields; (o) Ptosis; (p) Transient visual loss (monocular or binocular); (q) Symptomatically stable established patient with afferent visual pathway disorder (IIH, compressive optic neuropathy etc.)
			Prior to the COVID-19 pandemic, were you performing formal remote interpretation of diagnostic testing? (e.g., formal interpretation of fundus photography, visual fields, OCT without having a face-to-face encounter with the patient) (For U.S. members, use reference date of before March 1, 2020)	(a) Yes; (b) I could, but I still opted to see the patient in person for whom I interpret testing; (c) No
			Prior to the COVID-19 pandemic, approximately how many formal remote interpretations of diagnostic testing did you perform weekly? (For U.S. members, use reference date of before March 1, 2020)	Numerical response
			Prior to the COVID-19 pandemic, what types of diagnostic testing were you performing formal remote interpretation of? (For U.S. members, use reference date of before March 1, 2020) select all that apply	(a) Fundus photography; (b) visual fields; (c) Optical coherence tomography; (d) Visual evoked potentials; (e) ERGs; (d) Other (fill in)
			Presently, do you perform formal remote interpretation of diagnostic testing as of (e.g., fundus photography, visual fields, OCT)?	(a) Yes, this is part of my practice; (b) I can, but I still see that patient in person for whom I am interpreting; (c) No
			Presently, approximately how many formal remote interpretations of diagnostic testing did you perform weekly?	Numerical response
			Presently, what types of remote diagnostic testing are you formally interpreting? (Check all that apply)	(a) Fundus photography; (b) visual fields; (c) Optical coherence tomography; (d) Visual evoked potentials; (e) ERGs; (d) Other
			Do you plan to continue remote interpretation of diagnostic testing after the COVID-19 public health emergency subsides?	(a) Yes; (b) No; (c) Unsure
			Prior to the COVID-19 pandemic, did you provide fee-for-service asynchronous telemedicine “second opinions,” defined as record-based review without direct interaction with the patient, including a report to the patient? (For U.S. members, use reference date of before March 1, 2020)	(a) Yes; (b) No—my institution does not allow it; (c) No—other reason
			Presently, do you presently provide fee-for-service asynchronous telemedicine second opinions, defined as record-based review without direct interaction with the patient, including a report to the patient?	(a) Yes; (b) No—my institution does not allow it; (c) No—other reason
			Prior to the COVID-19 pandemic, did you provide and bill for interprofessional consultations without direct interaction with the patient with a follow-up verbal or written report to the referring doctor? (For U.S. members, use reference date of before March 1, 2020, these would include CPT codes 99451 or 99446–99449)	(a) Yes; (b) No
			Presently, do you provide and bill for interprofessional consultations without direct interaction with the patient with a follow-up verbal or written report to the referring doctor? (For U.S. members, use reference date of before March 1, 2020, these would include CPT codes 99451 or 99446–99449)	(a) Yes; (b) No
Sieberer (2022)^20^	No	7	Did you feel the patients had sufficient explanation and instruction on the approach of the consultation?	(1) Very poor/strongly disagree; (2) Poor/disagree; (3) Good/agree; (4) Very good/strongly agree
			Would you have wished for nurses to give a more detailed instruction on the line of action?	(1) Very poor/strongly disagree; (2) Poor/disagree; (3) Good/agree; (4) Very good/strongly agree
			Did you feel you were able to take history adequately?	(1) Very poor/strongly disagree; (2) Poor/disagree; (3) Good/agree; (4) Very good/strongly agree
			Did you feel safe throughout the consultation and examination?	(1) Very poor/strongly disagree; (2) Poor/disagree; (3) Good/agree; (4) Very good/strongly agree
			Did you feel you were able to carry out examination adequately?	(1) Very poor/strongly disagree; (2) Poor/disagree; (3) Good/agree; (4) Very good/strongly agree
			Did you feel you had enough room to meet patient’s concerns and discuss their diagnosis and treatment options?	(1) Very poor/strongly disagree; (2) Poor/disagree; (3) Good/agree; (4) Very good/strongly agree
			How do you feel supported by clinical staff (nurses/receptionists)?	(1) Very poor/strongly disagree; (2) Poor/disagree; (3) Good/agree; (4) Very good/strongly agree
			How do you feel about the new approach overall?	(1) Very poor/strongly disagree; (2) Poor/disagree; (3) Good/agree; (4) Very good/strongly agree
			I liked.../Positive comments	Open ended
			I did not like.../Areas of improvement	Open ended
Summers (2022)^22^ Pediatrics	No	61	I received the training needed to effectively implement telehealth visits.	(a) Strongly disagree; (b) Disagree; (c) Neither agree nor disagree; (d) Agree; (e) Strongly agree
			The training sessions were organized and provided adequate time on presented topics	(a) Strongly disagree; (b) Disagree; (c) Neither agree nor disagree; (d) Agree; (e) Strongly agree
			The training sessions encouraged discussion and feedback	(a) Strongly disagree; (b) Disagree; (c) Neither agree nor disagree; (d) Agree; (e) Strongly agree
			I received clearly described protocols to implement telehealth visits	(a) Strongly disagree; (b) Disagree; (c) Neither agree nor disagree; (d) Agree; (e) Strongly agree
			I have the skills needed to effectively implement telehealth visits	(a) Strongly disagree; (b) Disagree; (c) Neither agree nor disagree; (d) Agree; (e) Strongly agree
			The amount of time, money, and energy needed to implement the telehealth visits is reasonable	(a) Strongly disagree; (b) Disagree; (c) Neither agree nor disagree; (d) Agree; (e) Strongly agree
			The implementation team for my department (clinicians, PAS, technicians/orthoptists, photographers) share responsibility for the success of the telehealth visits	(a) Strongly disagree; (b) Disagree; (c) Neither agree nor disagree; (d) Agree; (e) Strongly agree
			The implementation team members from my department have defined roles and responsibilities	(a) Strongly disagree; (b) Disagree; (c) Neither agree nor disagree; (d) Agree; (e) Strongly agree
			Telehealth visits are supported by leadership	(a) Strongly disagree; (b) Disagree; (c) Neither agree nor disagree; (d) Agree; (e) Strongly agree
			I know where to get help if I encounter problems with telehealth visits	(a) Strongly disagree; (b) Disagree; (c) Neither agree nor disagree; (d) Agree; (e) Strongly agree
			The tools provided through Epic/OHSU supported my ability to implement telehealth	(a) Strongly disagree; (b) Disagree; (c) Neither agree nor disagree; (d) Agree; (e) Strongly agree
			The tools provided through Casey supported my ability to implement telehealth	(a) Strongly disagree; (b) Disagree; (c) Neither agree nor disagree; (d) Agree; (e) Strongly agree
			I feel confident in my ability to effectively implement telehealth visits	(a) Strongly disagree; (b) Disagree; (c) Neither agree nor disagree; (d) Agree; (e) Strongly agree
			I trust the results/findings from telehealth visits	(a) Strongly disagree; (b) Disagree; (c) Neither agree nor disagree; (d) Agree; (e) Strongly agree
			I think telehealth visits will improve outcomes for our patients	(a) Strongly disagree; (b) Disagree; (c) Neither agree nor disagree; (d) Agree; (e) Strongly agree
			I think telehealth visits have advantages for patients	(a) Strongly disagree; (b) Disagree; (c) Neither agree nor disagree; (d) Agree; (e) Strongly agree
			I prefer having telehealth visits over clinic visits	(a) Strongly disagree; (b) Disagree; (c) Neither agree nor disagree; (d) Agree; (e) Strongly agree
			Please rank the importance of the following items from 1–10. 1 being the most important and 10 being the least important for successful implementation	(a) Equipment and materials; (b) Communication to patients; (c) Patient interest; (d) Staff buy-in; (e) Clinician buy-in; (f) Leadership buy-in; (g) Plans for periodic patient outcome monitoring; (h) Plans for periodic review of telehealth delivery by clinical leadership; (i) Training; (j) Working together

AAO, American Academy of Ophthalmology; AMA, American Medical Association; AMD, age-related macular degeneration; ASRS, American Society of retina specialists; CNV, choroidal neovascularization; DR, diabetic retinopathy; EHR, electronic health record; EMR, electronic medical record; ERGs, electronic retinal grading system; HIPAA, Health Insurance Portability and Accountability Act; IIH, idiopathic intracranial hypertension; MR, magnetic resonance; MeSH, medical subject heading; OCT, optical coherence tomography; OHSU, Oregon Health and Science University; OKN, optokinetic nystagmus; OPD, outpatient department; PAS, patient administration system; ROP, retinopathy of prematurity; VOR, vestibulo ocular reflex.

## Results

### Survey subjects

The final dataset used included 22 studies. Four (18.2%) surveyed both providers and patients, 8 (36.4%) surveyed the opinions of providers only, 9 (40.9%) surveyed patients only, and 1 (4.5%) surveyed the parents of pediatric patients regarding a pediatric teleophthalmology visit. Most of the studies directed at providers surveyed ophthalmologists, but some included optometrists or ophthalmology staff members. Four studies elicited the opinions of specific subspecialists within ophthalmology, including 2 surveys of neuro-ophthalmologists (365 total respondents),^15,30^ 1 of oculoplastic surgeons (70 respondents),^27^ and 1 of vitreoretinal surgeons (214 respondents).^31^ In total, these 22 studies included 3,732 patient/parent participants and 2,388 provider respondents (Tables 1 and 2). We note that one study did not specifically report the total number of patient survey participants, but we used the N values listed for specific questions to estimate the total responses.^22^

### Validated questionnaires

Among the 22 included studies, four (18.2%) specifically note that they utilized externally validated questionnaires.^14,19,25,26^ Three of the studies utilizing validated surveys were performed in the United States and assessed patient perspectives,^14,19,25^ and one was performed in India and assessed provider perspectives.^26^ The authors of one additional publication note that their patient-directed survey was internally validated by use on 10 study subjects, but did not undergo external validation.^24^

One of the validated patient studies utilized the Preferences for Telemedicine Questionnaire,^19^ a 14-item survey that includes 5 yes/no questions assessing telemedicine knowledge followed by 9 questions assessing telemedicine beliefs, concerns, and predispositions on a 5-point Likert scale.^32^ Another used a modification of the RAND teleophthalmology questionnaire to assess the attitudes of diabetic patients regarding remote screening for diabetic retinopathy.^25^ The third study^14^ utilized a questionnaire adapted from the Telehealth Satisfaction Scale, a 10-item survey with responses on a 4-point Likert scale initially developed to assess specialist access via telehealth among First Nation peoples^33^ and validated in the memory clinic setting.^33^ In total, these 3 patient-directed studies utilizing validated questionnaires contained 103 questions after excluding demographics and patient data questions. After removal of the patient data and demographics questions, the validated surveys contained 11,^14^ 22,^19^ and 69^25^ question prompts, whereas the nonvalidated surveys ranged in length from 4 to 10 questions. A total of 459 patients responded to these 3 surveys.

The only provider-directed survey noting validation, Agarwal et al., surveyed 1,026 providers using a 25-item questionnaire, which was prevalidated by 4 independent ophthalmologists and pilot tested via use with 20 providers.^26^ This study was performed in India and contained the largest group of provider respondents of the included publications. The main focus of the Agarwal et al. study was to assess the financial impact of the COVID-19 pandemic on ophthalmology practices in India, and only one question in the survey directly addressed attitudes toward teleophthalmology. This question read “What is your experience regarding utility of teleophthalmology in your practice during lockdown period and after (Multiple options can be marked), with 6 answer options reading “No experience with teleophthalmology,” “It did not match my expectation and not many of my patients were benefitted,” “It benefitted my patients, and I am satisfied,” “It was billed similar to my outpatient department (OPD) face-to-face consultation charges,” “It was billed lower than my usual consultation charges,” and “It was not billed.”^26^

### Nonvalidated questionnaires

The remaining 18 studies included in the final dataset (81.8%) used nonvalidated surveys constructed by the authors, with significant variation in question style and response format. The authors of three of these studies did note that their questions had been adapted from separate validated surveys, but had not been validated for use in the setting of teleophthalmology.^18,22,34^ These surveys demonstrated significant variety in question wording, response wording, and response style (Likert, yes/no, numerical score, open ended, multiple choice).

### Patient surveys

We identified several themes among the patient-directed survey questions, including preexisting knowledge of telemedicine, satisfaction with remote care, willingness to use teleophthalmology in the future, and feelings of safety during the examination (Table 1). Five questions asked about confidentiality, and two asked whether the patient felt safe during their teleophthalmology encounter, one of these specifically in the setting of COVID-19. Seven patient questions asked about the timing of the visits: two regarding the amount of time spent during the encounter with testing or with the ophthalmologist, three regarding waiting time, one asked satisfaction with the time it took to obtain results, and one asked whether the teleophthalmology encounter reduced the time required to travel to a clinic. Three specifically asked about the convenience of receiving eye care remotely compared with in-person, and five asked whether the patient would recommend a teleophthalmology visit to others. Two questions asked about cost, both on a 5-point Likert scale:

(1) “To what extent do you agree with these statements: telemedicine could contribute to reducing health care system costs,”^19^ and
(2) “Did teleophthalmology save the cost of medical expenses?^23^”

### Provider surveys

Twelve publications included questionnaires directed at providers (Table 2). Common themes included provider utilization of telemedicine, home monitoring, or any remote care, including remote imaging interpretation, especially in the context of the COVID-19 pandemic. Twenty-three questions included reference to cost, overhead, or reimbursement for telehealth visits. Several questions assessed the providers’ views on the adequacy and reliability of the examinations, such as “Were you able to complete an examination as part of the virtual health visit that provided enough information for medical decision-making?,”^15^ “Did you feel you were able to carry out examination adequately?,”^20^ and “I trust the results/findings from telehealth visits.”^22^

Only one provider question specifically included the term “satisfaction,” stating “Score, on a scale from 0 to 10, your degree of general satisfaction with the activity.”^17^ Fourteen questions asked whether the provider planned to continue telemedicine visits, remote image reading, or home monitoring activities in the future, whereas 13 questions asked about barriers encountered in providing remote eye care visits. Prompts assessing the providers’ opinions on the utility of teleophthalmology for patients and their ocular health outcomes included:

(1) “Do you think that online video calls/examination applications are beneficial to the health service received by the patient?,”^35^
(2) “In your opinion, is telemedicine an effective tool for oculoplastic consultations?,”^27^
(3) “In its current form, I find telemedicine visits to be an acceptable form of evaluation for retina patients in the right clinical scenario,”^28^
(4) “I think telehealth visits will improve outcomes for our patients,”^22^ and
(5) “I think telehealth visits have advantages for patients.”^22^

## Discussion

Telemedicine has been slow to catch hold in ophthalmology despite its successful implementation in other health care fields. This discussion is especially prudent in the context of a growing shortage of ophthalmology providers in the United States,^36^ particularly in rural areas,^29^ and in the wake of the global COVID-19 pandemic. Telemedicine has the opportunity to address these challenges but, in our opinion, has not been utilized to its potential. This review seeks to address a gap in knowledge regarding the patient and provider experience with teleophthalmology.

Overall, patients surveyed in these studies seem to be generally satisfied with remote eye care/teleophthalmology, and the majority indicated that they would like to continue to receive remote visits for eye care and would recommend the service to others. For example, 95% of 386 patients in India agreed with the statements “I would use the mobile clinic again,” and “I would recommend the mobile clinic to others.”^37^ In a separate study out of Spain, 99.1% of 114 patients responded Good or Very Good when asked about “the feeling of being in good hands during the test.”^17^

There was variation in provider responses regarding satisfaction. When asked in the study by Conway et al., “Were you able to complete an examination as part of the virtual health visit that provided enough information for medical decision-making?,” 137/157 (87.3%) providers replied “Yes.”^15^ However, in a separate study conducted by Mercer et al., only 28/63 (45%) responded with Modest/Large when asked about their perception of the “role for teleophthalmology in future practice.”^38^ The Agarwal et al. study from India asked providers, “What is your experience regarding the utility of teleophthalmology in your practice during the lockdown period and after?,” and 326/780 (41.7%) responded with the option “It benefited my patients, and I am satisfied.”^26^

While these data are intriguing, it is challenging to summarize given the variability in question and answer design across the studies. Many extracted questions addressed the same underlying theme, but with different wording, highlighting a lack of standardization. For example, consider the following questions asked of patients from 4 different studies:

(1) “Do you feel that the virtual health visit satisfied your needs, or did it feel like you still needed to be seen in-person?,”^15^
(2) “Score, on a scale from 0 to 10, for degree of general satisfaction with the activity,”^17^
(3) “Thinking about your recent telehealth consultation, how would you rate your overall satisfaction?,”^21^
(4) “Did you feel that your examination has been adequate?”^20^

All four items address general satisfaction with tele-eye care from the patient’s perspective; however, the variation in wording and answer styles makes the data impossible to compile for increased statistical power. Response choices among the extracted questions included Likert scales, binary yes/no, multiple choice, numerical scales, and open ended.

The predominant use of author-developed surveys rather than validated questionnaires for assessing teleophthalmology is consistent with the trend found in other reviews of telemedicine surveys.^39–41^ Recent literature provides support for the development of a validated and standardized tool with which to assess telemedicine encounters. Such a tool could enhance the accuracy of evaluating patient-centered aspects of audiovisual telemedicine interactions and contribute to the development and harmonization of standardized assessment frameworks across telehealth platforms and medical domains.^42,43^ However, it is important to acknowledge that such an instrument may not fully reflect the unique characteristics of specific programs, geographic regions, or population groups. Using validated surveys is especially beneficial in increasing the power of pooling studies with limited participants; in this case, the largest patient cohort was 1,079,^44^ whereas the largest provider cohort was 1,026.^26^ Some of these studies addressed subspecialized areas within ophthalmology: of the 22 studies examined, 2 addressed neuro-ophthalmology^15,30^ with a total of 365 providers surveyed, 2 were specific to glaucoma with 1,189 patients total,^19,44^ 7 addressed diabetic retinopathy/retina,^16,17,25,28,31,34,45^ 1 oculoplastics with 70 providers,^27^ and 1 pediatric ophthalmology with 89 parents surveyed.^21^ Given the small number of studies and participants in each of these subgroups, utilizing validated surveys with the ability to pool cohorts would be even more beneficial for identifying trends. Some subspecialties within ophthalmology may be more amenable to virtual care than others, such as oculoplastics, where remote visits may be a reliable method for assessing eyelid position and orbital abnormalities. In contrast, neuro-ophthalmologists may find it challenging to obtain a reliable pupil and extraocular movement examination virtually, but these studies are small overall and have low power.

Another consideration is that these 22 studies utilized differing methods of delivering remote eye care. For example, some telemedicine protocols required patients to present in-person to an office for ocular imaging, followed by asynchronous evaluation by offsite ophthalmologists. Other studies used live video visits or telephone encounters without any direct patient contact. Some included studies did not directly state how the visits were performed within their methods or did not provide a detailed description of the setup. The lack of standardization in the care delivery methods makes the results difficult to combine and interpret. While some patients/providers may strongly favor video visits, their answers may differ when trying phone visits instead, but with the current published studies, these differences are difficult to determine. Additional factors such as network reliability and platform usability may be important in patient and provider experience with teleophthalmology, but were not discussed in many of these studies.

We note several limitations to this study. Demographic data about the providers and patients could provide useful insight, but variation in the way these data were collected between studies made it difficult to pool and report. In addition, a rating of study quality could be useful for future studies. However, assessing study quality based on design is challenging because it involves multiple nuanced factors such as sample size, methodology, and bias control. These elements vary by context and cannot be easily captured in a single score, making expert judgment essential.

Telemedicine has not become a mainstream practice in ophthalmology, although it is difficult to pinpoint the exact reasons. Studies assessing teleophthalmology have increased since the onset of the COVID-19 pandemic, with 18 of the 22 studies included in this review published in 2020 or later. Despite a growing interest in remote delivery of medical care postpandemic, further investigation into teleophthalmology is needed before it can become widely implemented, and generalized conclusions are only possible if small studies can be pooled to have higher power. Here, we review the landscape of published studies assessing patient or provider opinions on teleophthalmology through direct surveys, identifying trends where evident. The development of standardized guidelines and evaluation metrics specific to teleophthalmology would be a worthwhile goal for national and international ophthalmological organizations, to draw evidence-based conclusions about the utility of teleophthalmology and promote its implementation.

## Data Availability

Additional data available from the corresponding author upon reasonable request.

## Abbreviations Used

AAO: American Academy of Ophthalmology
AMA: American Medical Association
AMD: Age-related macular degeneration
ASRS: American Society of retina specialists
CNV: Choroidal neovascularization
DM: Diabetes mellitus
DR: Diabetic retinopathy
EHR: Electronic health record
EMR: Electronic medical record
ERGs: Electronic retinal grading system
HIPAA: Health Insurance Portability and Accountability Act
IIH: Idiopathic intracranial hypertension
MR: Magnetic resonance
MeSH: Medical subject heading
OCT: Optical coherence tomography
OHSU: Oregon Health and Science University
OKN: Optokinetic nystagmus
OPD: Outpatient department
PAS: Patient administration system
RAND: RAND corporation
ROP: Retinopathy of prematurity
VOR: Vestibulo ocular reflex
